# *KRAS* Mutational Profiles among Colorectal Cancer Patients in the East Coast of Peninsular Malaysia

**DOI:** 10.3390/diagnostics13050822

**Published:** 2023-02-21

**Authors:** Hidayati Husainy Hasbullah, Sarina Sulong, Nur Asyilla Che Jalil, Ahmad Aizat Abdul Aziz, Nurfadhlina Musa, Marahaini Musa

**Affiliations:** 1 Human Genome Centre, School of Medical Sciences, Universiti Sains Malaysia, Kota Bharu 16150, Malaysia; 2 Department of Pathology, School of Medical Sciences, Universiti Sains Malaysia, Kota Bharu 16150, Malaysia

**Keywords:** colorectal cancer, *KRAS*, codons 12 and 13, precision medicine

## Abstract

Background: *KRAS* is a key driver gene in colorectal carcinogenesis. Despite this, there are still limited data on the mutational status of *KRAS* amongst colorectal cancer (CRC) patients in Malaysia. In the present study, we aimed to analyze the *KRAS* mutational profiles on codons 12 and 13 amongst CRC patients in Hospital Universiti Sains Malaysia, Kelantan, located on the East Coast of Peninsular Malaysia. Methods: DNA were extracted from formalin-fixed, paraffin-embedded tissues obtained from 33 CRC patients diagnosed between 2018 and 2019. Amplifications of codons 12 and 13 of *KRAS* were conducted using conventional polymerase chain reaction (PCR) followed by Sanger sequencing. Results: Mutations were identified in 36.4% (12/33) of patients, with G12D (50%) being the most frequent single-point mutation observed, followed by G12V (25%), G13D (16.7%), and G12S (8.3%). No correlation was found between mutant *KRAS* and location of the tumor, staging, and initial carcinoembryonic antigen (CEA) level. Conclusion: Current analyses revealed that a significant proportion of CRC patients in the East Coast of Peninsular Malaysia have *KRAS* mutations, where this frequency is higher compared to those in the West Coast. The findings of this study would serve as a precursor for further research that explores *KRAS* mutational status and the profiling of other candidate genes among Malaysian CRC patients.

## 1. Introduction

Colorectal cancer (CRC) is the third most commonly diagnosed cancer worldwide and it is one of the leading causes of cancer death, second only to lung cancer [1]. By 2030, CRC cases are predicted to increase by 60% to more than 2.2 million new cases and 1.1 million deaths [2]. In Malaysia, CRC is the most common cancer diagnosed in males and second in females according to the Malaysia National Cancer Registry 2012–2016 [3]. The majority of CRC cases are diagnosed at the advanced stage of malignancy. This contributes to the high morbidity and mortality due to this disease.

The global burden of CRC necessitates the development of a novel biomarker that aims to improve the detection, management, and/or treatment outcome of patients diagnosed with this cancer. One such marker is *KRAS* proto-oncogene, GTPase. Recent studies have shown that *KRAS* mutational status is a robust negative predictive marker for response to anti-epidermal growth factor receptor (EGFR) monoclonal antibodies, namely cetuximab and panitumumab. These EGFR-directed therapies are now routinely used as first-line and subsequent-line therapy for metastatic CRC in combination with standard chemotherapy. 

Here, *KRAS*, known as Kirsten rat sarcoma viral oncogene homolog, is a member of the RAS type GTPase family of genes. The gene encodes *KRAS* protein, a small GTPase transductor protein, located downstream from EGFR in the RAS/RAF/MEK/ERK pathway. This pathway is one of the most important mitogen-activated protein kinase (MAPK) signaling pathways involved in cell proliferation and differentiation [4]. Mutations in *KRAS* result in EGFR-independent activation of the MAPK pathway by reducing the GTPase activity. This will lead to uncontrolled cell proliferation, potentially occurring in the early stages of colorectal carcinogenesis, as postulated in the chromosomal instability (CIN) pathway [5]. The CIN pathway is the most important pathway for the development of CRC, representing up to 80% of sporadic CRC cases. Mutation in *KRAS* is proposed to drive the progression of a small adenoma to a large adenoma before further genetic alterations result in the development of carcinoma and its progression. 

To date, more than 90% of the activating *KRAS* mutations identified in CRC occurred at codons 12 and 13 in exon 2 of the gene. Single base substitution of glycine to aspartate (G12D) and glycine to valine (G12V) on codon 12 are the most common mutations observed [6,7]. Although mutations at exons 3 and 4 have also been recorded, these accounted for only about 1–4% of *KRAS* mutations [6,8,9].

The *KRAS* mutational rates among CRC patients vary according to the population studied, ranging from 30 to 52% [10,11,12]. The explanation for the differences in the prevalence of *KRAS* mutations among CRC patients across the globe is uncertain. In Malaysia, the prevalence is lower (approximately 20%), as previously reported [13,14,15]. Despite this, *KRAS* remained as one of the most common genes mutated in CRC patients in Malaysia [16]. These previous studies, however, were limited to CRC patients who underwent surgical treatment in Kuala Lumpur and Selangor. The population demographic of these two states on the West Coast of Peninsular Malaysia is strikingly different from those on the East Coast. Kuala Lumpur, for instance. has a population comprised of approximately 40% Malays, 40% Chinese, and 10% Indians. In contrast, more than 90% of Kelantanese are Malays, while Chinese and Indians encompass less than 5% of the population, respectively [17]. Another state in the East Coast of Peninsular Malaysia, Terengganu, also has a similar demographic and ethnic group distribution. Murad et al. (2012) [13] reported that *KRAS* mutations were found more commonly in Chinese patients than in other ethnicities, and this may be due to differences in the genetic susceptibility toward CRC. 

Cases of CRC continue to rise in Kelantan, with an increment of 16% of new cases reported from 2012–2016 in comparison to previous data from 2007–2011 [3]. Recent data from the Endoscopy Unit and Pathology Department showed that around 6% of patients who underwent colonoscopy from January to August 2020 were subsequently diagnosed with CRC or colonic adenoma (unpublished data) at Hospital Universiti Sains Malaysia (USM), which is one of the main public hospitals in Peninsular Malaysia.

To the best of our knowledge, no published local study has yet explored the *KRAS* mutational status among CRC patients from the East Coast of Peninsular Malaysia. Knowing the mutational status of this important gene in CRC among patients in this region is central to developing background data on the pattern of gene mutations involved in Malaysian CRC patients. In this era where precision medicine and targeted therapy is the current treatment goal for cancer, the findings of this study may also provide clinicians and pathologists with the necessary input in the development of relevant genetic testing as part of a diagnostic workup for CRC patients in this region in the near future. 

## 2. Materials and Methods

Ethical clearance was obtained from the Human Research Ethics Committee of USM (USM/JEPeM/21030249) before the commencement of this study. 

### 2.1. Participants

A total of 33 formalin-fixed-paraffin-embedded (FFPE) tissue samples were obtained. These specimens were collected from CRC patients who underwent colon and/or rectal resection surgery with confirmed histopathology diagnosis of CRC in Hospital USM from January 2018 to December 2019. Detailed information regarding the clinicopathological and demographic data of each patient including age, gender, ethnicity, histological differentiation, location of the primary tumor, nodal status, distant metastasis, treatment received, and American Joint Committee on Cancer (AJCC) stage were collected from the medical records and laboratory information system (LIS) of the Hospital USM. Exclusion criteria were (1) a family history of hereditary colorectal carcinoma, (2) multiple primary malignancies, (3) previous treatment with anti-EGFR monoclonal antibodies, (3) histology showing conditions other than adenocarcinoma, and (4) secondary cancer which metastasized to the colon. Images of hematoxylin and eosin (H&E)-stained slides of selected cases were acquired from the Department of Pathology, USM, and reviewed by the pathologist.

### 2.2. DNA Extraction from CRC FFPE Tissues

Genomic DNA was extracted using a commercial QIAamp^®^ DNA FFPE Advanced Kit (Qiagen, Hilden, Germany), according to manufacturer’s instructions. Quantification of the extracted DNA was performed using Infinite® M200 NanoQuant (Tecan Group Ltd., Mannedorf, Switzerland). 

### 2.3. Detection of Mutations in Codons 12 and 13 of KRAS 

**Figure 1:**
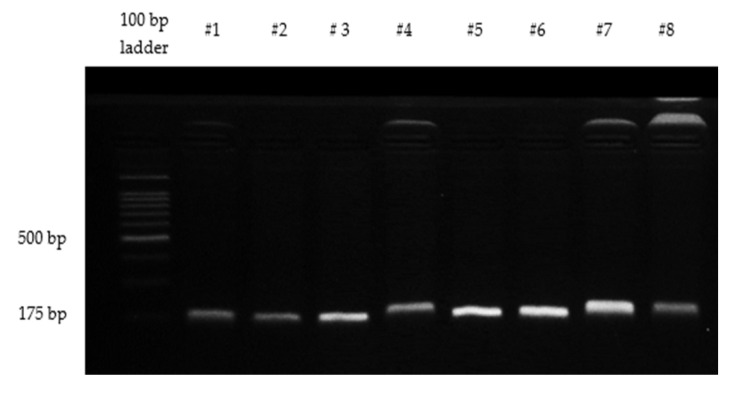
Gel electrophoresis image of selected PCR products targeting codons 12 and 13 of *KRAS*.

For amplification of codons 12 and 13 of *KRAS*, polymerase chain reaction (PCR) was performed on all extracted DNA samples using Agilent SureCycler 8800 Thermal Cycler (Agilent Technologies, Inc., Santa Clara, CA, USA). The primer sequences (5′–3′) were adopted from previous study [14]. The forward primer sequence used was ACCTTATGTGTGCATGTTC while the reverse primer sequence used was CTATTGTTGGATCATATTCG. Cycling conditions were as follows: a pre-denaturing incubation at 95 °C for 2 min, 35 cycles of 95 °C for 30 s, annealing temperature of 50 °C for 30 s, and 72 °C for 30 s followed by a final extension at 72 °C for 5 min. Each PCR reaction contained 100 ng genomic DNA, 2.5 mM MgCl2, 0.20 mM of each deoxynucleotide triphosphate (dNTP), 0.2 µM of each primer, and 1U of *Taq* DNA polymerase, in a final reaction volume of 20 µL. The PCR product quality was confirmed by gel electrophoresis using 2% agarose gel, run at 90 volts for 45 min. A distinct band at 175 bp was produced for each PCR product, corresponding to the primers used (Figure 1). Purification of the PCR product and DNA sequencing were performed by a commercial company (Apical Scientific Sdn. Bhd., Selangor). The DNA chromatograms and sequences were examined using the BioEdit^®^ v7.2.3 Sequence Alignment Editor (Informer Technologies, Inc., Los Angeles, CA, USA) and the BLAST sequence analysis tool, respectively. Tumors with *KRAS* mutations were classified as mutant *KRAS*, while those without *KRAS* mutations were classified as wild-type *KRAS*. 

### 2.4. Statistical Analysis

Results were presented as frequency (*n*) and percentage (%) for categorical variables. Comparison between groups was made using the chi-squared test or Fisher’s exact test. Statistical analysis was performed using IBM SPSS Statistics V27.0 (IBM Corp., Armonk, NY, USA) software. Statistical significance was set at a *p*-value of 0.05 or less. 

## 3. Results

### 3.1. Patient Clinical Characteristics

The clinical characteristics of the participants are summarized in Table 1, according to *KRAS* mutation status. 

**Figure 2:**
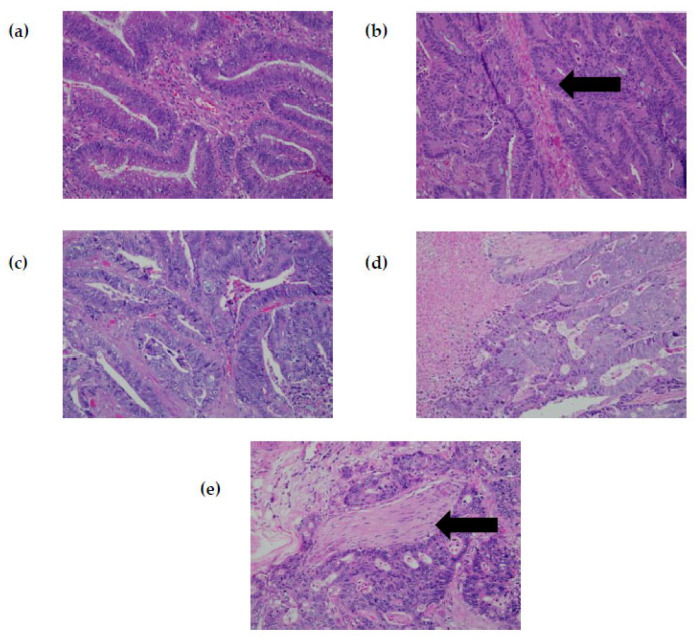
Histological examinations (H&E stain, 200×) in selected cases. (**a**) Well-differentiated adenocarcinoma in Case 1 (G12D mutation), showing tumor cells arranged in an irregular glandular pattern infiltrating the stroma; (**b**) well-differentiated adenocarcinoma in Case 2 (G12S mutation), exhibiting malignant glands arranged mainly in a complex glandular pattern which infiltrates into the muscularis propria (black arrow); (**c**) moderately differentiated adenocarcinoma in Case 11 (G13D mutation). Surrounding stroma shows a marked desmoplastic reaction; (**d**) moderately differentiated adenocarcinoma in Case 15 (wild-type *KRAS*) with area of necrosis; (**e**) moderately differentiated adenocarcinoma in Case 19 (wild-type *KRAS*) exhibiting a cribiform pattern with perineural invasion (black arrow).

The samples were collected from 18 (54.5%) female and 15 (45.5%) male CRC patients. The median age of the participants was 62 years (range 29–84). A total of 84.8% (*n* = 28) of the patients were of Malay ethnicity while the remaining 15.2% (*n* = 5) were Chinese. No patients from Indian or other ethnicities were documented in this study. Majority of recruited patients have primary tumors located on the left side of the colon (*n* = 25, 75.8%) whereas only 3 (9.1%) cases had right-sided CRC and 5 (15.2%) patients had rectal tumors. Pathological staging analysis of the tissues showed that nearly 70% (*n* = 23) of the samples were classified at either stage III or IV of the malignancy. Most of the CRC samples were moderately differentiated adenocarcinoma (*n* = 27, 82%) while one sample was noted to be a mucinous adenocarcinoma subtype. Figure 2 shows representatives of the H&E-stained slides from selected patients. The median initial serum CEA was 20.4 ng/mL (range 1.9–914.8). A positive CEA level, defined as a value of >5 ng/mL was observed at initial diagnosis in 24 (72.7%) of the participants. 

### 3.2. Mutation Characteristic of KRAS

**Figure 3:**
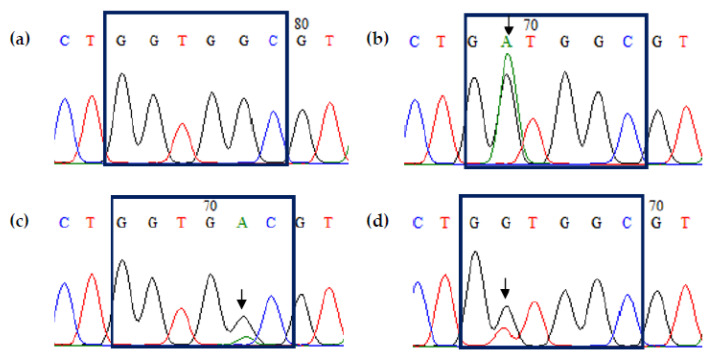
Chromatogram highlighting codons 12 and 13 of *KRAS* in selected cases. Arrows indicate the location of the single-point mutations. (**a**) Wild-type *KRAS* sequence (GGT GGC); (**b**) c.35G > A (G12D) mutation in case 4; (**c**) c.38G > A (G13D) mutation in case 9; (**d**) c.35G > T (G12V) mutation in case 12.

Among the 33 tumor samples tested, 12 (36.4%) had *KRAS* mutations at the respective codons: 83.3% (10/12) were single-point mutations at codon 12, while 16.7% (2/12) mutations were observed at codon 13. The most common point mutation detected was G12D (6/12, 50%), followed by G12V (3/12, 25%), G13D (2/12, 16.7%), and G12S (1/12, 8.3%) (Table 2). Figure 3 represents the DNA sequencing analysis of codons 12 and 13 of the *KRAS* gene in selected cases.

### 3.3. Relationship between Clinicopathological Features of CRC and KRAS Mutations

As shown in Table 1, *KRAS* mutations were not associated with gender, age, and ethnicity of the CRC patients studied (*p* = 0.469; *p* = 0.392; *p* = 0.630, respectively). No correlation was found between mutant *KRAS* and the location of the tumor, staging, and initial CEA level (*p* = 0.420; *p* = >0.950; *p* = >0.950, *p* = 0.429, respectively).

Table 3 shows that liver (9/13, 69%) and lung (8/13, 62%) are the two most common sites of distant metastases in all stage IV CRC cases, while 38% (5/13) had both liver and lung involvement. All advanced CRC *KRAS*-mutant patients had lung metastases (5/5, 100%) while only half of stage IV wild-type *KRAS* patients (4/8, 50%) had lung involvement. The clinicopathological features of patients with mutant *KRAS* are summarized in Table 4.

## 4. Discussion

Various strategies can be employed for the detection of mutations in *KRAS* including Sanger sequencing, pyrosequencing, and ARMS/Scorpion real-time PCR. We use Sanger sequencing as it is an established method for mutation analysis and also due to the limited availability of equipment and expertise in our laboratory. Although the overall sensitivity of direct sequencing is modest in comparison to more recent techniques, the rate of detection of *KRAS* mutations has been shown to be increased in specimens with advanced CRC [18].

Using direct sequencing, we identified that a significant proportion of CRC patients (12/33, 36.4%) had mutations in codon 12 or 13 of *KRAS*. This finding is consistent with various published reports [8,9,19]. Interestingly, our data indicated that CRC patients in the East Coast of Malaysia had a higher *KRAS* mutation rate compared to those from the West Coast (36.4% vs. 22%) [13,14,15]. Murad et al. (2012) [13] suggested that *KRAS* mutations were seen more commonly in the Chinese population compared to in Malays. However, in the present study, Malay patients constituted the majority of the participants (84.8%) and there was no significant association between ethnicity and *KRAS* mutational status (*p* = 0.630). Current finding suggested that more than race, other aspects, such as environment, diet, or lifestyle factors, may play a significant and prominent role in the acquirement of *KRAS* mutations in CRC. These observations are in agreement with reports by Cefalì et al. (2021) [20], who noted that *KRAS* mutations were observed more frequently in African Americans compared to in Africans living in their native country (37% vs. 21%). In addition, a study conducted in Mexico demonstrated that the *KRAS* mutation rate varies from 40% in the North Pacific region to 59% in the central Mexican region [20,21]. Both studies attributed the discrepancy in *KRAS* mutation rates to different dietary cultures between the studied groups. Residents in the North Pacific region of Mexico, for instance, consumed a diet less heavy in meat compared to those in the central Mexican region.

In the Malaysian population, it was established that smoking, red meat intake, and a high fat, high energy, and low fiber diet have a significant association with the risk of CRC [22,23]. However, the environment, dietary patterns, and lifestyles of Malaysian citizens vary according to geographical location. The Malaysian National Health and Morbidity Survey 2019 [24] reported that the prevalence of adult obesity and inadequate intake of vegetables was higher in Kelantan state compared to in Kuala Lumpur and Selangor states. None of the literature explicitly explores red meat consumption and other CRC high-risk foods or nutrient intakes (i.e., heme iron and fat) among residents in different regions of Malaysia. In one study, high levels of vegetable intake were associated with a reduced risk of *KRAS* mutations [25]. The protective effect of vegetables against *KRAS* mutation is believed to be due to the richness of fiber and bioactive compounds that act to prevent the formation of nitroso compounds in the intestine. Nitroso compound is known to induce guanine base alkylation, which can lead to G to A base substitution in *KRAS* if not repaired [26]. However, a systematic review exploring the associations between nutritional factors and *KRAS* mutations showed highly conflicting and inconsistent results [26]. Further research, preferably a prospective study design using a bigger sample population exploring these factors and twin studies, is necessary to support our observation.

The spectrum of *KRAS* mutations in the present study is also in accordance with published data. The most common *KRAS* mutation occurred at codon 12 (83.3%) with G12D (50%) and G12V (25%) being the two most frequent single-point mutations observed, followed by G13D (16.7%). Various studies are being conducted exploring the true prognostic role of a specific codon mutations in CRC but, to date, no consensus has been obtained [11,27,28].

Recently, G12C mutation gained considerable interest after the FDA approved the use of sotorasib, a *KRAS*-G12C protein inhibitor, in metastatic non-small cell lung carcinoma (NSCLC) with G12C mutation. In CRC, the benefit of sotorasib is still under evaluation, but has shown considerable potential [29]. The G12C mutation is seen in approximately 2–4% of CRC patients, but was lacking in the present study cohort, possibly due to small sample size.

Our results showed that there is no significant association between *KRAS* mutational status and location of the primary tumor. This finding is in agreement with multicentered RASCAL I and RASCAL II collaborative studies and various published reports [10,30,31]. However, recent studies by Xie et al. (2019) [32], which include meta-analysis of the previous literature, demonstrated that right-sided CRC has a significantly higher rate of mutated *KRAS*. The correlation between tumor sidedness and *KRAS* mutations is highly controversial. The inconsistent findings across different studies in the literature may be partly due to a lack of uniformity in the definition of the right-sided and left-sided colon. Some studies categorized rectum under the left-sided CRC [12,33]. Similarly, tumors located at the transverse colon may be categorized separately or included under right-sided CRC. We separated the rectum from left-sided CRC, as the rectum represents a separate anatomical and topographical entity with a different risk for carcinogenesis compared to colonic mucosa [34].

It is widely accepted that right-sided and left-sided colon cancer has a distinct molecular carcinogenesis. This distinctive genetic makeup may be attributed to differences in embryonic origin, microbial load, and some discrete physiological functions of the right and left colon [35]. Right-sided colon cancer commonly exhibits the microsatellite instability-high (MSI-H) tumor subtype, while left-sided colon cancer is characterized by mutations in the CIN-related pathway [36,37]. Sporadic MSI-H tumors develop due to defects in the mismatch repair (MMR) genes, for example hyper-methylation of MSH1, or mutations in other MMR genes, such as *MSH6*, *MSH2*, and *MLH3*. On the other hand, CIN tumors are characterized by various copy number variants (CNV) in the tumor tissue, caused by aneuploidy, insertions, deletions, amplifications, and loss of heterozygosity (LOH). The genes commonly involved in CIN carcinogenesis include *APC*, *KRAS*, and *TP53*. Although in a theory proposed by Fearon and Vogelstein in 1990, the adenomas progress to carcinomas in a sequential of events involving loss or mutation of *APC*, mutation of *KRAS,* and mutation of *TP53*, recent studies have shown that only 6.6% of CRC cases have mutations involving all these three driver genes [38]. Taken together, our findings suggest that *KRAS* mutation is not indicative of CRC tumor location, despite being proposed as part of the CIN carcinogenesis pathway.

We included all stages of CRC in our study, with stage I and II cases categorized together to represent early-stage CRC. We noted that around 70% of cases were at the advanced stage of the malignancy at diagnosis, in accordance with national data [3]. The high proportion of CRC patients with advanced diseases corresponds to the low uptake of CRC screening among the general Malaysian population. A huge study exploring the uptake for CRC screening in various Asian countries revealed that only 1.2% Malaysian respondents went for CRC screening, in contrast to Singapore (20.3%), Thailand (18.8%), and Brunei (8.8%) [39]. In the Malaysian National Strategic Plan for Colorectal Cancer (2021–2025) [40], the Health Ministry aim to improve the screening rate of the target age group by increasing the screening coverage from 10.8% to 40.0%, and to subsequently decrease the CRC cases by 25% by the year 2030.

In addition to that, we discovered that there were no significant differences in the distribution of *KRAS* mutations across all stages. Our finding is consistent with various published reports [8,10]. This may suggest that *KRAS* is not involved in the progression of CRC and that other driver genes are responsible for the development of advanced CRC. Yuen et al. (2002) [41] showed that the *KRAS* mutation rates were similar in both sporadic colorectal adenoma and carcinoma, supporting the role of *KRAS* in early CRC tumorigenesis. On another spectrum, various reports have also shown that there is no preponderance for certain types of *KRAS* mutations during the metastatic process of CRC, indicating that *KRAS* is not crucial in the acquirement of metastatic ability [42]. Furthermore, the concordance rate of *KRAS* mutation status between the primary CRC tumor and its corresponding metastases is high, supporting the notion that these mutations are acquired before the dissemination of the tumor cells to distant organs [43].

We noted that amongst the 13 patients with metastatic CRC (mCRC), 38.5% of cases had *KRAS* mutations, corresponding to published data. Interestingly, in this present study cohort, all advanced CRC patients with mutated *KRAS* had lung metastases, while only half of wild-type *KRAS* patients had lung involvement. Various studies had demonstrated that the lung is the most common site of distant metastases in *KRAS*-mutated CRC, compared to other solid organs, such as the liver and brain [9,43]. However, the explanation for this observation remains unclear.

Our data also revealed that there is no correlation between mutant *KRAS* and initial CEA level (*p* = 0.429), although the majority of our mutant *KRAS* patients presented with positive CEA at diagnosis. Wojciechowicz et al. (2000) [44] demonstrated that *KRAS* mutant cells expressed significant CEA levels in their colorectal cell culture studies. However, clinical research exploring their associations showed conflicting results. A study by Selcukbirik et al. (2003) [45] suggested a significant correlation between *KRAS* mutations and high initial CEA level, while Zhao et al. (2021) [46] claimed no differences in CEA levels between wild-type and mutant *KRAS* in CRC. The laboratory test for CEA is simple, inexpensive, and typically uses an established automated assay. Various studies explored its association with *KRAS* mutations to determine if CEA levels are predictive for a *KRAS*-mutant tumors, but most studies are against its predictive role.

Our present study focuses only on *KRAS* mutational status and does not include other biomarkers that also predict lack of response to anti-EGFR therapy, such as *NRAS* and *BRAF*. Follow-up studies exploring the presence of mutations within these genes—especially among the wild-type *KRAS* patients—are desirable.

It is worth mentioning that our results need to be interpreted with caution, as the present study is susceptible to potential biases due to its small sample size. This is a major limitation of the current study and is due to the limited resources and time available. Further studies with participation from more patients across different health institutions within the East Coast of Peninsular Malaysia are suggested to increase the robustness of this current finding. Future analyses of *NRAS* and *BRAF* would also benefit from increased sample sizes, as mutations within these genes are present at a low frequency among CRC patients. Our study did not explore *KRAS* mutations at other codons, for example codon 61 or 146. Although codons 12 and 13 represent the majority of the known, associated *KRAS* mutations in the CRC, there is a possibility that our data may underestimate the true *KRAS* mutational rate in the study cohort. Despite these limitations, we believe that present study has provided the necessary background information regarding the *KRAS* mutational profiles among the local CRC patients in this region.

## 5. Conclusions

In conclusion, a significant proportion of CRC patients from the East Coast of Peninsular Malaysia have *KRAS* mutations. To the best of our knowledge, the present study is the first to demonstrate that the frequency of *KRAS* mutations among CRC patients is higher in our region compared to those in the West Coast of Peninsular Malaysia, as reported in the previous literature. Further study is warranted to explore the underlying factors that influence the difference in *KRAS* mutational rates between these two regions. The correlation between *KRAS* mutation status and clinicopathological features of CRC remains inconclusive despite extensive research to elucidate their associations. We reported that *KRAS* mutations are not associated with ethnicity, location and stage of the tumor, and initial CEA level. Clinically, *KRAS* is most useful as a predictive marker for response to anti-EGFR monoclonal antibodies, although it is anticipated to gain more significance in the future following the discovery of a *KRAS*-G12C protein inhibitor. The findings of this study would serve as a preliminary data for further research that explores *KRAS* mutational status and other candidate genes in the development and progression of CRC in a bigger sample and unique population of Malaysia.

## Figures and Tables

**Table 1:** Clinicopathological features of CRC patients according to *KRAS* mutational status.

Features	*KRAS* Status		*p*-Value
	Wild-Type *n* (%)	Mutant *n* (%)	
Total	21 (63.6)	12 (36.4)	
Gender Male Female	11 (73.3) 10 (55.6)	4 (26.7) 8 (44.4)	0.469
Age (years) ≤60 >60	9 (56.3) 12 (70.6)	7 (43.8) 5 (29.4)	0.392
Ethnicity Malay Chinese	17 (60.7) 4 (80)	11 (39.3) 1 (20)	0.630
Tumor site Right colon Left colon Rectum	3 (100) 15 (60) 3 (60)	0 (0) 10 (40) 2 (40)	0.420
Differentiation Well Moderate Poor	4 (57.1) 16 (64.0) -	3 (42.9) 9 (36.0) -	>0.950
AJCC Stage I or II III IV	7 (70) 6 (60) 8 (61.5)	3 (30) 4 (40) 5 (38.5)	>0.950
CEA (ng/mL) ≤5 >5	7 (77.8) 14 (58.3)	2 (22.2) 10 (41.7)	0.429

**Table 2:** Frequency of *KRAS* mutations.

Mutation	Base Change	Frequency, *n* (%)
G12D	c.35G > A	6 (50)
G12V	c.35G > T	3 (25)
G12S	c.34G > A	1 (8.3)
G13D	c.38G > A	2 (16.7)

**Table 3:** Stage IV CRC with its corresponding metastatic site and *KRAS* mutation status.

Case	*KRAS* Mutation Status	Primary CRC Location	Corresponding Metastatic Site(s)
3	G12D	Left colon	Liver, Lung, Spine
5	G12V	Left colon	Lung
6	G12D	Left colon	Liver, Lung
9	G13D	Left colon	Lung, Adrenal
11	G13D	Left colon	Lung
14	Wild-type	Left colon	Liver
16	Wild-type	Left colon	Lung
19	Wild-type	Left colon	Liver, Lung
23	Wild-type	Left colon	Liver, Peritoneum
24	Wild-type	Rectum	Liver
26	Wild-type	Right colon	Liver, Lung
28	Wild-type	Left colon	Liver
30	Wild-type	Rectum	Liver, Lung

**Table 4:** Characteristics of colorectal cancer patients with mutant *KRAS* and their clinicopathological features.

Case	Age (Years)	Race	Tumor			Initial CEA (ng/mL)	*KRAS* Mutations, Amino Acid Changes
			Site	Differentiation	Stage		
1	30	Malay	Rectum	Well	I	0.3	c.35G > A, G12D
2	72	Malay	Left side	Well	III	3.8	c.34G > A, G12S
3	51	Chinese	Left side	Moderate	IV	914.8	c.35G > A, G12D
4	58	Malay	Left side	Moderate	III	12.6	c.35G > A, G12D
5	71	Malay	Left side	Moderate	IV	28.1	c.35G > T, G12V
6	39	Malay	Left side	Moderate	IV	166.9	c.35G > A, G12D
7	82	Malay	Left side	Well	I	12.2	c.35G > A, G12D
8	40	Malay	Left side	Moderate	III	66.5	c.35G > A, G12D
9	60	Malay	Left side	Moderate	IV	173.2	c.38G > A, G13D
10	83	Malay	Rectum	Moderate	III	25.0	c.35G > T, G12V
11	78	Malay	Left side	Moderate	IV	276.1	c.38G > A, G13D
12	59	Malay	Left side	Moderate	II	27.0	c.35G > T, G12V

## Data Availability

Data obtained and analyzed during the study are available from the corresponding author upon request. The data are not publicly available as they contained information that may compromise the privacy of the participants.
